# Effective and new technologies in kidney tissue engineering

**DOI:** 10.3389/fbioe.2024.1476510

**Published:** 2024-10-16

**Authors:** Hossein Rayat Pisheh, Mobin Haghdel, Mahboube Jahangir, Monireh Sadat Hoseinian, Shaghayegh Rostami Yasuj, Ali Sarhadi Roodbari

**Affiliations:** ^1^ Department of Tissue Engineering and Applied Cell Sciences, School of Advanced Medical Sciences and Technologies, Shiraz University of Medical Sciences, Shiraz, Iran; ^2^ Student Research Committee, Shiraz University of Medical Sciences, Shiraz, Iran

**Keywords:** tissue engineering, scaffold, 3D bioprinting, microfluidic systems, kidney cells, kidney diseases

## Abstract

Kidney disease encompasses a wide spectrum of conditions, ranging from simple infections to chronic kidney disease. When the kidneys are unable to filter blood and remove waste products, these abnormalities can lead to kidney failure. In severe cases of kidney failure, kidney transplantation is considered the only definitive treatment. Worldwide, the World Health Organization (WHO) repeatedly emphasizes the importance of organ donation and increasing transplantation rates. Many countries implement national programs to promote the culture of organ donation and improve patient access to kidney transplantation. The extent to which this procedure is performed varies across countries and is influenced by several factors, including the volume of organ donation, medical infrastructure, access to technology and health policies. However, a kidney transplant comes with challenges and problems that impact its success. Kidney tissue engineering is a new approach that shows promise for repairing and replacing damaged kidney tissue. This article reviews recent advances in kidney tissue engineering, focusing on engineered structures such as hydrogels, electrospinning, 3D bioprinting, and microfluidic systems. By mimicking the extracellular environment of the kidney, these structures provide suitable conditions for the growth and development of kidney cells. The role of these structures in the formation of blood vessels, the mimicry of kidney functions and the challenges in this field were also discussed. The results of this study show that kidney tissue engineering has high potential for treating kidney diseases and reducing the need for kidney transplantation. However, to achieve clinical application of this technology, further research is required to improve the biocompatibility, vascularization and long-term performance of engineered tissues.

## Graphical Abstract

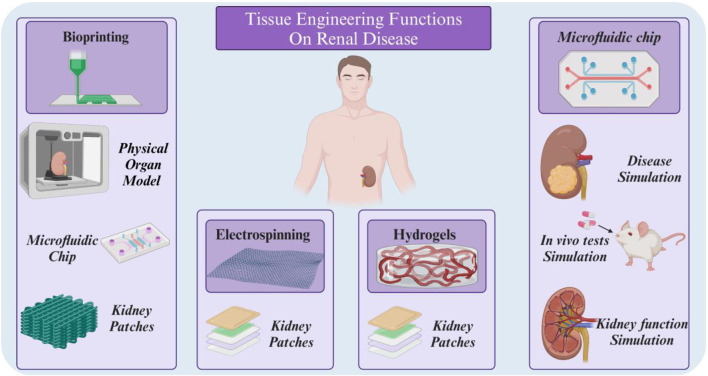

## 1 Introduction

The kidney is a visceral and complex organ of the body composed of more than 20 types of specialized cells (Borges and Schor, 2018). Damage to this organ leads to diseases with acute and chronic kidney symptoms (Hinze et al., 2024). A sudden increase in serum creatinine concentration, often accompanied by a decrease in urine production, is one definition of acute kidney injury (AKI). AKI indicates a series of pathological changes such as necrosis and apoptosis of renal tubules, changes in the filtration barrier, defective glomerular filtration, vasoconstriction and tubular obstruction, as well as interstitial swelling and activation of proteolytic enzymes (Nourie et al., 2024; Yang et al., 2024). On the other hand, in chronic kidney disease (CKD), damaged kidney cells are not replaced by functional tubular cells, leading to fibrosis and tubulointerstitial scarring (Du et al., 2023; Ni et al., 2024). Current treatment for AKI and CKD is limited to lifelong dialysis or kidney transplantation, which has resulted in significant improvement in kidney function. Although dialysis can replace the function of kidney filtration by removing some toxins from the blood, this method cannot restore many other functions of the kidney, such as the production of erythropoietin (EPO) and the activation of vitamin D. These limitations, along with the physical complications associated with dialysis, can lead to a poor quality of life, high mortality, and long-term survival problems (Cohen, 2020; Katagiri et al., 2021; Tang et al., 2024). Kidney transplantation is also considered an effective treatment for chronic kidney failure. However, this method faces challenges and limitations that exist worldwide. These challenges include the lack of organ donations, safety issues (transplant rejection and infection), high transplant costs, unequal distribution of resources, and age and comorbidities (Nagendra et al., 2023; Robinson et al., 2021).

**FIGURE 1:**
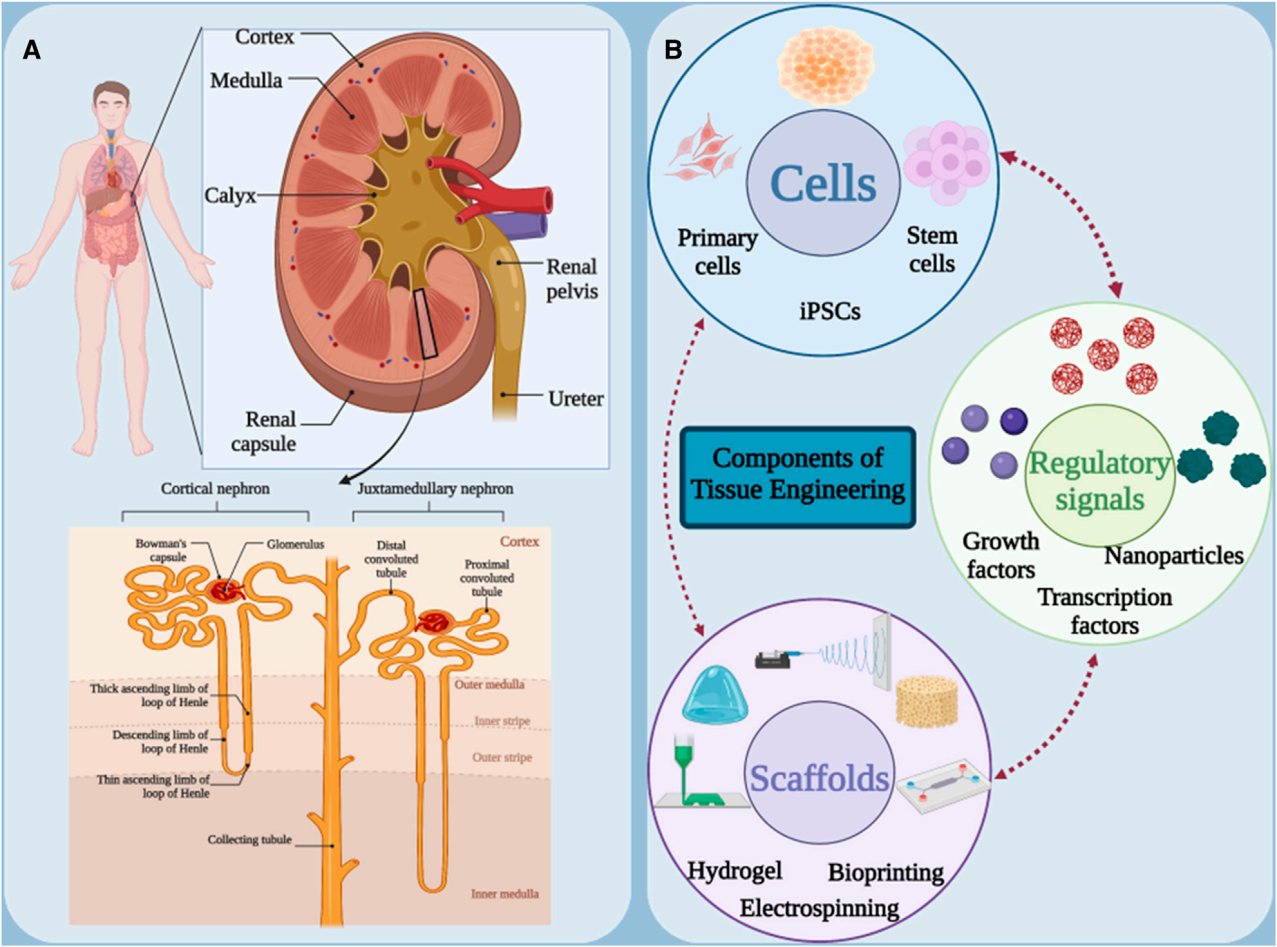
**(A)** Schematic of the anatomical structure of the kidneys. **(B)** Main components in tissue engineering. Figure was created with BioRender.com.

In order to be able to offer a suitable treatment method, it is necessary to know the structure and functional units of the kidney. Kidneys are two bean-shaped, brown organs located on either side of the spine behind the abdominal cavity and at the level of the twelfth thoracic to third lumbar vertebrae. Because of its proximity to the spleen, the left kidney is slightly higher than the right kidney. These two vital organs play a very important role in maintaining body homeostasis. Each kidney is surrounded by a sturdy fibrous capsule that protects it. The functional part of the kidney or parenchyma is divided into two main parts, the renal cortex and the renal medulla (Figure 1A) (Makishima et al., 2024; Paksuz, 2023). The renal cortex is the outer part of the kidney and contains the glomeruli, the initial part of the nephron tubules and the proximal convoluted tubules. The renal medulla is the inner part of the kidney and contains the loops of Henle and the collecting ducts (Chang and Davies, 2019; Lopez-Gonzalez et al., 2020). The medulla is divided into renal pyramids, the tip of which points towards the renal pelvis. The renal pelvis is a cave-like space that collects urine produced in the nephrons and directs it to the ureter (Davies et al., 2021). Nephron is the functional unit of the kidney and consists of glomerulus, Bowman’s capsule, proximal convoluted tubule, loop of Henle, distal convoluted tubule and collecting duct (Kumaran and Hanukoglu, 2020; Palmer and Schnermann, 2015). Kidneys have three main functions: glomerular filtration, tubular reabsorption and tubular secretion. During glomerular filtration, blood enters the glomerulus through the afferent artery, and its liquid part (filtrate) is filtered into Bowman’s capsule. In tubular reabsorption, useful substances such as water, glucose, amino acids and ions are reabsorbed from the nephron tubes and return to the blood, and in tubular secretion, waste substances such as urea, creatinine and drugs are excreted from the blood into the nephron tubes. Blood enters the kidney via the renal artery and leaves via the renal vein. The kidneys are innervated by sympathetic and parasympathetic nerves (Gronda et al., 2023; Nishi et al., 2015; Osborn et al., 2021; Zhang and Mahler, 2023).

In order to apply a suitable treatment method, the damaged kidney in AKI and CKD must be regenerated with functional kidney-specific cells, which suggests new cell-based approaches to replace the damaged kidney cells. Tissue engineering has been proposed as a promising solution to solve the mentioned problems by developing tissue-engineered kidney structures with normal kidney function. The development of therapies based on tissue engineering strategies has created several alternative options for the treatment of renal pathologies at different levels of the disease. Tissue engineering is an emerging field of medical science that aims to restore, replace or improve the function of damaged body tissue. This method attempts to create new kidney tissue or restore function to damaged tissue by mimicking the body’s natural tissue processes. For this purpose, three main elements of scaffolding, cells and signaling (growth factors) are used (Figure 1B) (Cui et al., 2023; Golchin et al., 2023). Scaffolds are three-dimensional structures that serve as a framework for cell growth. They are usually made of natural or synthetic materials and must have properties such as appropriate porosity, biocompatibility and biodegradability (Soleymani Eil Bakhtiari and Karbasi, 2024). Kidney cells can be obtained from various sources, such as stem cells, kidney epithelial cells or kidney fibroblast cells. These cells are cultured on a scaffold to form new tissue (Mahdavi and Enderami, 2022). Finally, signaling molecules stimulate the growth and differentiation of cells and help form tissue or restore function (Zyuz’kov, 2022). Kidney tissue engineering can replace damaged kidneys, reduce the need for kidney transplants, treat congenital kidney disease, and model kidney disease. In general, kidney tissue engineering is an innovative and promising approach to the treatment of kidney diseases, which can help improve patients’ quality of life and reduce the burden of disease.

In this review, we have focused on four key technologies within the field of tissue engineering (TE): hydrogels, electrospinning, 3D printing, and organ-on-chip. Our selection of these specific areas is based on their significant contributions to the advancement of TE and their potential for addressing critical unmet clinical needs. Hydrogels have emerged as a versatile and biocompatible platform for tissue engineering due to their ability to mimic the extracellular matrix (ECM) and provide a supportive environment for cell growth and function. Unlike spheroids or polymeric scaffolds, hydrogels offer a high degree of tunability, allowing for precise control of their mechanical properties, degradation rates, and biological signaling cues (Lee and Hwang, 2023). Also, Electrospinning, a technique for creating nanofibrous scaffolds, has gained significant attention in TE for its ability to generate structures that closely resemble the ECM. These scaffolds provide a high surface area-to-volume ratio, promoting cell adhesion, migration, and differentiation (Zulkifli et al., 2023).

3D printing has revolutionized TE by enabling the fabrication of complex and customizable tissue constructs with precise control over their architecture and composition. This technology offers the potential to create patient-specific tissues and organs, addressing the limitations of traditional transplantation methods (Mahendiran et al., 2021). Organ-on-chip systems, which integrate microfluidic technology with cellular and tissue models, have emerged as powerful tools for studying tissue-level interactions and physiological processes. These systems offer a more physiologically relevant environment compared to traditional *in vitro* models, facilitating drug discovery and personalized medicine (Du et al., 2024).

Focusing on these four technologies, we aim to provide an overview of the current state of TE research and highlight promising avenues for future development, and based on this knowledge, recent advances in the treatment of kidney diseases are reviewed.

## 2 Hydrogels

Hydrogels are three-dimensional networks made of water-soluble polymers that are physically or chemically cross-linked to form a gel (Wang et al., 2023). Hydrogels have unique properties such as their soft biomechanical similarity to natural soft tissue, adjustable pores, biocompatibility, suppression of inflammatory reactions and easy functionalization. By imitating the body’s extracellular matrix, these substances provide a suitable environment for the growth and proliferation of cells (Lu et al., 2024). Hydrogels are used in medical research for a variety of purposes, including cell carriers, scaffolds to improve cell adhesion or proliferation, fillers to fill defects and promote healing, or drug delivery systems (Lee and Hwang, 2023). To design hydrogel scaffolds, physicochemical properties (such as degradation, cross-linking, porosity, diffusion, etc.) and biological properties should be designed according to the target tissue and the type of intended function (Kallingal et al., 2023).

**FIGURE 2:**
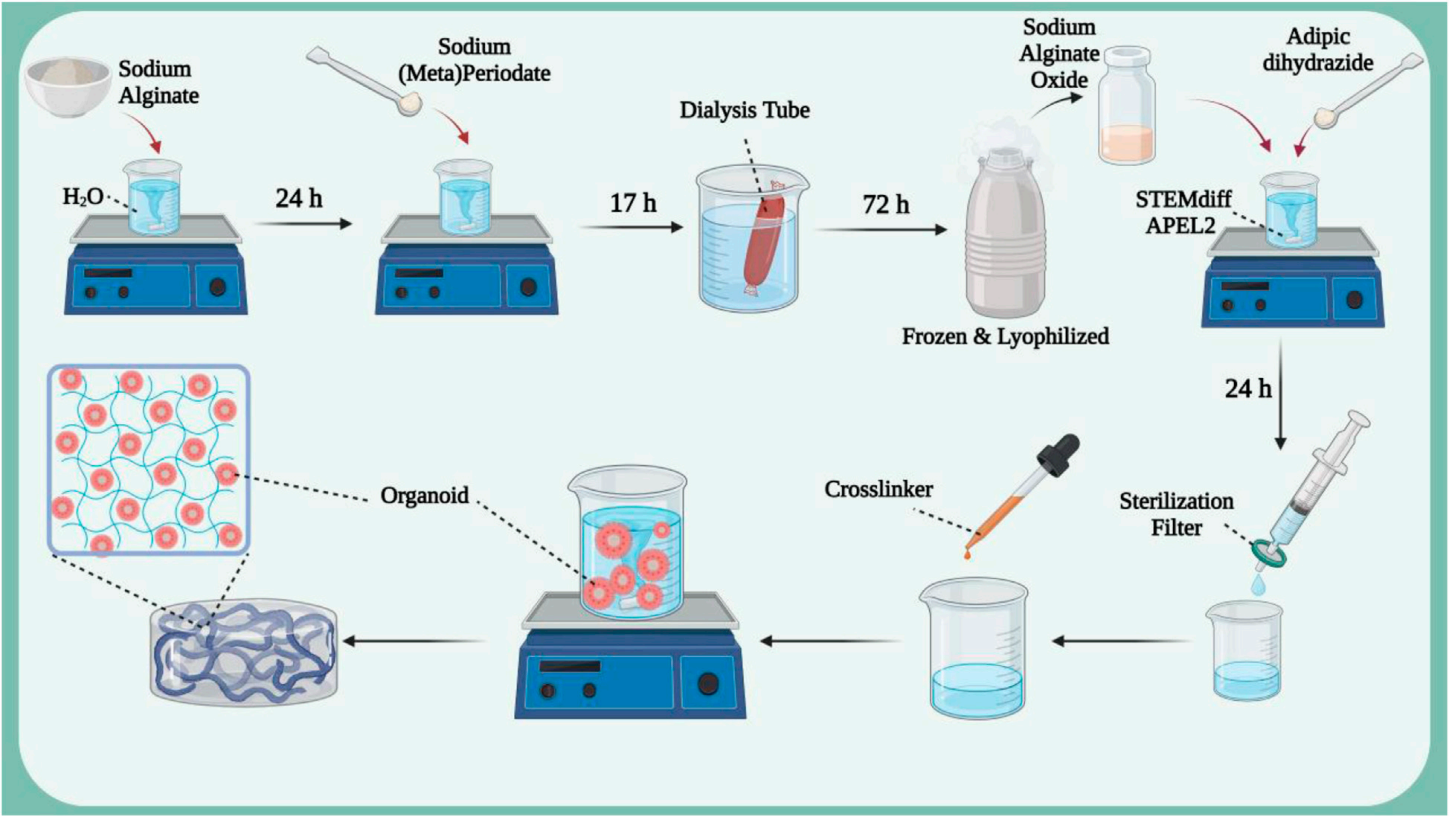
The synthesis process of hydrogel based on sodium alginate oxide in which kidney organoids derived from human pluripotent stem cells are loaded. Figure was created with BioRender.com.

Hydrogel architectures have different dynamic properties. This property can be exploited to create a controlled and biomimetic environment for organoid encapsulation, as differentiation in hydrogel environments is fully controllable and physiologically relevant to improve organoid reproducibility and maturation (Ruiter et al., 2022). Kidney organoids derived from human pluripotent stem cells offer a promising solution to kidney failure, but often result in off-target cells and phenotypic changes that prevent maturation. Therefore, there is a need to modify the biophysicochemical parameters that control the formation of kidney organoids (Ruiter et al., 2022; Treacy et al., 2023; Yu et al., 2023). One study examined the effect of hydrogel stiffness on renal phenotype and adverse fibrotic markers (Figure 2) (Ruiter et al., 2022). In this study, pure sodium alginate was dissolved in deionized H_2_O and then added to a sodium (meta)periodate solution. The resulting product was dialyzed in NaCl solution for 3 days and then frozen in liquid N_2_ and lyophilized to obtain sodium alginate oxide. A hydrogel was then fabricated from the resulting material to encapsulate the organoid. The results showed that encapsulation in soft, supportive hydrogels resulted in the formation of all major kidney segments, polarization of apical proximal tubules and formation of primary cilia, as well as less fibrosis or EMT-related proteins. In a similar study, a hydrogel was synthesized using decellularized kidney extracellular matrix (dECM) to culture hPSC-derived kidney organoids (Kim et al., 2022). The results of creating an extensive vascular network have been reported. Single-cell transcription revealed that cultured kidney organoids have more mature glomerular growth patterns and more closely resemble human kidneys. Transplantation of this hydrogel to rat kidney showed that it accelerates the absorption of endothelial cells from the kidney of the host rat and the integration of vessels with more organized diaphragmatic gap structures.

**FIGURE 3:**
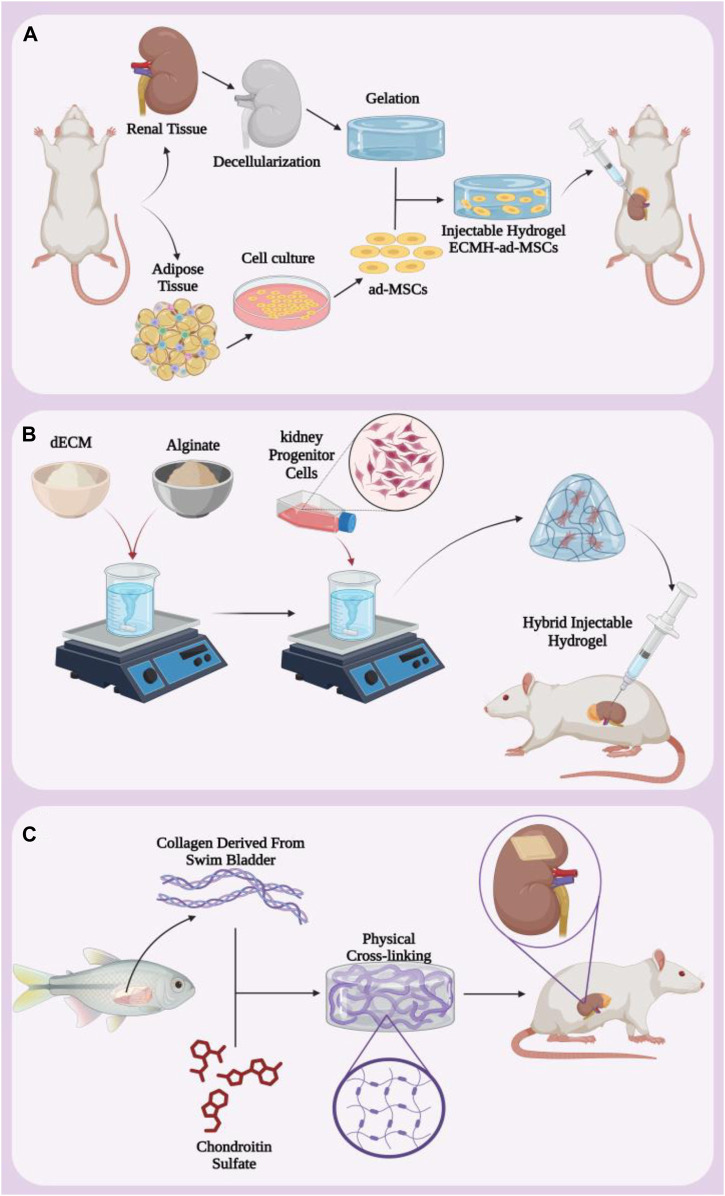
**(A)** Protocol for making hybrid injectable hydrogel containing ECMH and ad-MSCs. **(B)** Injectable hydrogel containing dECM and alginate that carries kidney progenitor cells. **(C)** Making kidney hydrogel patch from collagen extracted from fish swim bladder and chondroitin sulfate. Figure was created with BioRender.com.

Due to their unique properties mentioned above, hydrogels can be produced in a wide range of shapes and sizes. These three-dimensional three-dimensional structures can be produced in the form of bulk gels, microgels, nanogels, and hydrogel fibers in addition to films and patches, based on the intended application and goals. One of the most commonly used forms of hydrogels in treatment are injectable hydrogels, which can be injected into desired areas without the need for invasive surgery to apply patches or wipes. These hydrogels can contain cells, growth factors or drugs. Injectable hydrogels allow local delivery, which compared to systemic drug administration, is a unique alternative to increase efficacy, lower dose, and minimize systemic tissue toxicity, which is ideal (Bertsch et al., 2023; Gao et al., 2020). Renal ischemia/reperfusion (I/R) injury is a complex phenomenon that causes significant damage to renal tissue and can lead to acute renal failure and other serious complications. Mesenchymal cell-based therapy is a practical approach in the treatment of renal ischemia/reperfusion (I/R) injury, and the main challenge with this treatment modality is the low cell retention and survival at the ischemic site (Wu S. et al., 2022). To address this challenge, a study synthesized a hydrogel containing s-nitroso-n-acetylpenicillamine (SNAP) functionalized with RGD peptide bound to the integrin receptor and for the transplantation of Wharton Jelly mesenchymal stem cells (WJ-MSCs) evaluated (Najafi et al., 2022). The *in vivo* results showed that Fmoc-FF+Fmoc-RGD hydrogels supported the expansion and proliferation of WJ-MSCs *in vivo*, and intralesional injection of nitric oxide along with embedded WJ-MSCs resulted in better recovery after renal I/R injuries. The expression biomarkers of oxidative stress and nitric oxide synthase (iNOS) showed a significant decrease, causing protective effects such as reducing renal fibrosis and chronic inflammation. On the other hand, they observed increased expression of endothelial nitric oxide synthase (eNOS) and vascular endothelial growth factor (VEGF), indicating regeneration of endothelial tissue, reduction of cell death and inflammation, and improvement of damaged kidney function (Najafi et al., 2022). According to these results, it can be said that hydrogels containing WJ-MSCs can improve the regeneration of renal I/R injury by increasing angiogenic factors and cell transplantation, and it is an effective strategy to control and improve this injury. In another study for the treatment of renal I/R injury, an injectable hydrogel based on renal extracellular matrix was prepared to deliver adipose tissue-derived mesenchymal stem cells (ad-MSCs) (Zhou et al., 2020). Kidney extracellular matrix (ECM) is then prepared and lyophilized, dissolved in sterile water. The sediment obtained from the centrifuge is digested with pepsin enzyme and then diluted with phosphate buffer solution to obtain the pregel solution. Finally, after placing the solution at 37°C for 30 min, it turns into a hydrogel. Then ad-MSCs were added to the hydrogel (Figure 3A). The results of *in vivo* and *in vitro* analyzes showed that ECMH significantly increased the persistence and survival of mesenchymal stem cells in damaged kidneys. Furthermore, this hydrogel significantly reduced oxidative stress and apoptosis and, on the other hand, increased the proliferation, migration, secretion and epithelial differentiation of mesenchymal stem cells.

Injectable hydrogels are widely used for various therapeutic purposes due to their unique properties and high efficiency. In a study to evaluate renal progenitor cell delivery in kidney injury, they synthesized a hybrid injectable hydrogel based on decellularized renal extracellular matrix (dECM) and alginate (ALG) (Chu et al., 2022). First, solutions containing dECM and alginate were mixed together and then kidney progenitor cells were added. Finally, it was converted into a hybrid injectable hydrogel by physical cross-linking method in CaCl2 solution. It was then injected into the damaged kidneys of rats for *in vivo* assessments (Figure 3B). The addition of ALG to dECM increases the physical stiffness of the hydrogel and reduces the degradation of the hydrogel. Since dECM is collagen-based and ALG is a biodegradable polymer, this hydrogel showed remarkable biocompatibility and homeostatic behavior. In addition, at 7 and 21 days after injection, the glomerular structure was observed in the early stages and the phenomenon of a dense linear cell network and an increase in cell density due to the migration of cells from the host and the formation of a pattern were observed. The results showed that, in addition to having favorable physical properties to support damaged tissue during repair, dECM/ALG hydrogel is rich in progenitor cells, which may play a role in kidney regeneration in the long term. Despite the great and important progress of decellularized extracellular matrix-based scaffolds for kidney regeneration, several concerns and challenges regarding immune responses and complex composition still remain to be addressed.

Many researches have been done to simulate and bring the three-dimensional structure closer to the original body tissue in order to get the most efficiency from the hydrogel scaffold. For example, extracellular matrix simulating hydrogel scaffolds containing natural collagen (Col) derived from swim bladder and chondroitin sulfate (CS) derivatives were created as anti-fibrosis hydrogels (Wu et al., 2021). This hydrogel was synthesized through covalent and physical cross-linking (Figure 3C). The evaluation results showed that biomimetic hydrogels have mechanical properties, thermal stability and high biocompatibility both *in vitro* and *in vivo*. When this hydrogel is implanted in a semi-nephrectomized rat model, it increases the recruitment of native kidney cells and reduces tubular damage. This hydrogel induces the regeneration of renal tubular tissue and restores the metabolic function of the kidney. These results indicate that the biomimetic scaffold is a functional platform with high efficiency for the treatment of kidney diseases.

Choosing the right hydrogel shape is one of the most important steps in developing and manufacturing smart polymer materials. Taking into account the factors affecting the shape of hydrogels, it is possible to produce hydrogels with different properties and applications. Despite these mentioned advantages, the use of hydrogels in kidney tissue engineering is associated with challenges such as precise control of the mechanical properties of hydrogels, improving the blood supply (vascularization) of tissue engineering structures and extending the lifespan of these structures. However, with recent advances in materials, tissue engineering and cell biology, hydrogels are expected to improve and play an important role in the treatment of kidney diseases.

## 3 Electrospinning

**FIGURE 4:**
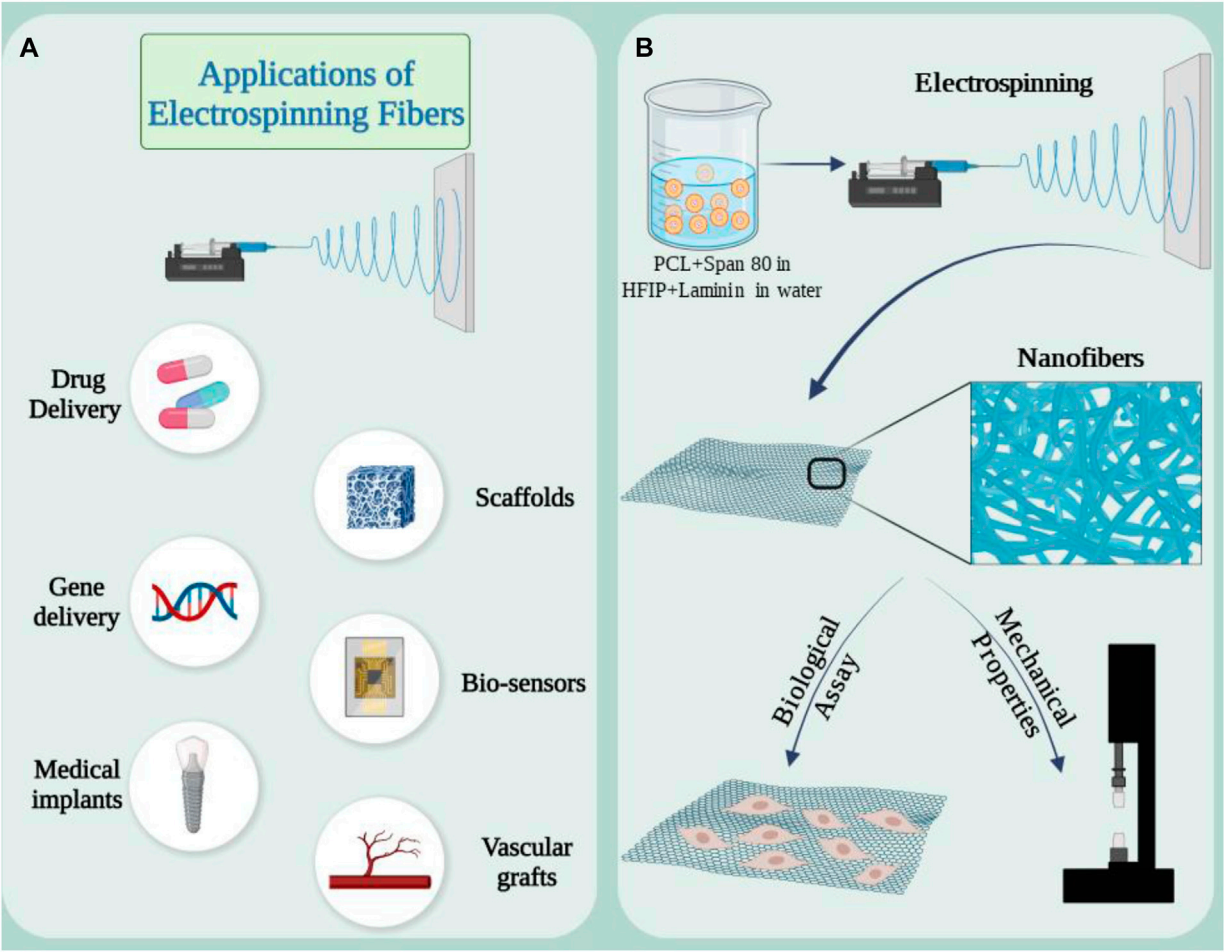
**(A)** Applications of electrospun nanofibers in medical research and development. **(B)** Synthesis of electrospun scaffold based on PCL and laminin emulsion and its capacity as a supporting substrate in kidney repair. Figure was created with BioRender.com.

Electrospinning is a scaffold fabrication technique that has been extensively studied in various tissue engineering techniques. This technology enables the production of continuous fibers in the nano and micro range from a wide variety of materials using different strategies (Huang T. et al., 2024). A strong electric field (in the kilovolt range) is applied to the solution at the tip of a nozzle. The applied force creates a Taylor cone due to the surface tension between the solution and the walls of the nozzle. When the applied electrical force overcomes the surface tension, a jet is formed from the Taylor cone to the collector and after evaporation of the solvent, solid fibers are formed (Jeong et al., 2022). In this technique, the diameter of the fiber can be adjusted by carefully controlling parameters such as nozzle size, applied electric field, polymer solution, etc. Fabricated nanofibers, have been of great interest in tissue engineering due to their topological dimensions, surface-to-volume ratio and high pore connectivity, as well as mimicking the morphology of the extracellular matrix of natural tissues. This technique has not yet been fully explored in kidney tissue engineering (Phutane et al., 2023; Younes et al., 2023). However, the advantages that make this technique attractive for kidney repair include biocompatibility, tunable mechanical properties, and the ability to release components needed for kidney tissue engineering, such as vascular endothelial growth factor (VEGF) (Braghirolli et al., 2017; Liu et al., 2023). Some applications and capacities of electrospinning scaffolds are shown in Figure 4.

Creating a niche where seed cells can act in the desired way can be a suitable strategy in using electrospinning scaffolds in kidney repair. The combination of extracellular matrix proteins with polymers can evaluate this effect in the kidney. In a study, an electrospun scaffold composed of polycaprolactone laminin was prepared to investigate this issue. In this method, scaffolds were enriched with laminin through direct combination with polymer solution or in the form of emulsion with a surfactant (Figure 4A) (Baskapan and Callanan, 2022). RC-124 cells, which are kidney epithelial cells, were cultured on the scaffold. The results showed that adding protein to the structure changed the mechanical properties. After 21 days of cell culture, biological evaluations were done. Gene expression analysis of healthy cells showed that E-CAD as a key marker for the formation of cell junctions in epithelial tissues was increased and upregulation could be a sign of monolayer formation. Collectively, data from key markers and physical properties show that the hybrid scaffolds support renal cells and provide a suitable environment during regeneration. Human induced pluripotent stem cell (hiPS) podocytes offer the opportunity to study development and kidney disease (Mou et al., 2022a). Studies on podocyte cells are carried out in the usual and traditional way in cell culture plates. This method does not reproduce the molecular and mechanical features required to model tissue surface functions, and overcoming these limitations requires the use of customizable and biomimetic scaffolds (Ozanne et al., 2024). Silk fibroin is suitable for regenerative applications due to its biocompatibility and versatility, including the ability to form electrospun fibers and membranes (Foppiani et al., 2024). In a study, it was shown that postmitotic hiPS cells can differentiate into renal glomerular podocytes on electrospun silk fibroin membranes containing laminin, and these differentiated cells exhibit high levels of podocyte-specific markers, consistent with the phenotype of mature cells (Mou et al., 2022a). This study demonstrated that electrospinning scaffolds can modify and alter the cultivation conditions according to the intended goals.

Electrospinning scaffolds are used in other strategies to repair the kidney or improve its function. In this study, a simple and innovative strategy to design a wearable artificial kidney using a new dialysis/absorption thin film nanofiber composite membrane (TFNC) composed of polyvinyl alcohol (PVA) and polyacrylonitrile was carried out (Ding et al., 2021). First, a PAN nanofiber membrane with UiO-66 (COOH)_2_ nanoparticles was fabricated. This membrane is a porous substrate with an affinity for effectively removing creatinine while significantly reducing dialysate volume in the dialysis process. The results of simulated dialysis evaluations showed that 62.8% of creatinine was removed in the simulation system and more than 98% of bovine serum albumin (BSA) passed through the membrane. Based on the results obtained, the introduction of the absorption function into the TFNC hemodialysis membrane provides the opportunity to develop a lightweight wearable artificial kidney that requires *in vivo* evaluations.

The metastasis of renal cell carcinoma is a major challenge and a solution needs to be found to solve this problem and improve the treatment effect. In a study, a controlled release system for paclitaxel as an anticancer drug was developed by fabricating a PLGA-silk protein nanofiber system (Cai et al., 2022). This nanofibrous structure was inspired by the occurrence and pathological microenvironment of renal cell carcinoma. The short-term release of paclitaxel can prevent the rapid spread and metastasis of kidney cancer, and the long-term and continuous release of the drug can prevent the proliferation of kidney cancer cells and recurrence. The drug delivery results showed that the silk protein nanofiber system enables relatively stable and long-term (1 month) release of paclitaxel. This drug delivery system can be a suitable platform for the controlled release of various drugs for the treatment of diseases or kidney dysfunction.

In this section, the high capacities and advantages of electrospinning in kidney tissue engineering were mentioned. These structures mimic the extracellular matrix (ECM) of native kidney tissues. Several studies demonstrated the potential of electrospun scaffolds to improve cell attachment, proliferation, and differentiation of kidney cells. However, several challenges remain, including the development of vascularization strategies to promote long-term survival of engineered kidney tissue. Overall, electrospinning is a versatile and powerful technique with great potential for advances in the field of kidney tissue engineering, raising hopes for kidney repair and improvement.

## 4 3D printing

3D printing is a new technology that has brought enormous changes in recent years in various fields, including medicine. 3D printing or additive manufacturing is a process in which a 3D object is created by joining successive layers of material together. This technology allows the creation of complex and personalized objects using 3D computer models (Ben Hadj Hassine et al., 2024; Franco Urquiza, 2024; Oleksy et al., 2023; Srivastava et al., 2023). One of the promising applications of this technology is in the field of tissue engineering and organ repair, especially the kidney. In recent years, 3D bioprinting has emerged as an innovative method for tissue engineering and organ repair. This promising technology enables the creation of living and functional tissues using patient cells, biomaterials and 3D printers. In this method, cells are placed into a biological ink along with biomaterials and then printed layer by layer onto a scaffold using a syringe or nozzle (Faber et al., 2023; Li et al., 2022). This framework acts as a matrix for the growth and reproduction of cells and is gradually replaced by new cells. Printed models of the kidney can be used to evaluate the effects of new drugs on kidney tissue, thereby accelerating the development of new drugs (Mayer et al., 2024; Yasen et al., 2024).

**FIGURE 5:**
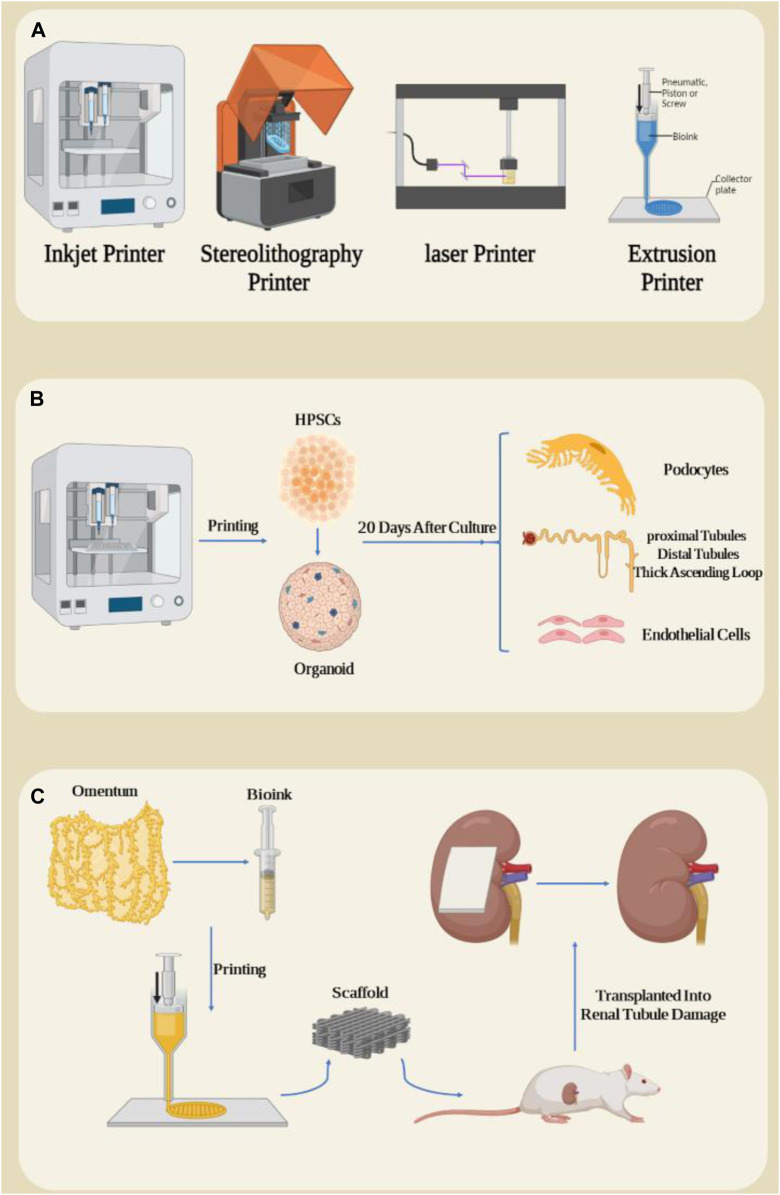
**(A)** Types of three-dimensional bioprinters. **(B)** 3D printing of HPSC cells and their organoid differentiation into kidney organoids, resulting in some important kidney structures such as Henle system after 20 days of organoid cultivation. **(C)** Preparation of a bioink from omentum and impression in the form of a kidney patch and transplantation in the form of a rat kidney subcapsule. Figure was created with BioRender.com.

Based on the working mechanism, there are different types of 3D bioprinters, including extrusion printers, inkjet printers, laser printers and stereolithography printers (Figure 5A) (Palaszko et al., 2024; Parthiban et al., 2021; Pavan Kalyan and Kumar, 2022; Prabhakaran et al., 2022; Xie et al., 2023; Zhang et al., 2023). Various factors such as the type of cells and biomaterials used, the required resolution, the complexity of the desired structure, the printing speed and the cost are crucial when selecting the type of biological 3D printer (Mir et al., 2023). Extrusion printers are the most common type and consist of bioink (a mixture of cells, biomaterials and growth factors) that is expelled through a printing nozzle and printed layer by layer onto a scaffold or substrate. The advantages of this method include compatibility with a variety of bioinks, precise control of extrusion volume and speed, and the ability to produce complex structures. However, its disadvantages include the possibility of nozzle clogging and limitations in printing high-resolution structures (Abdulmaged et al., 2022; Arjoca et al., 2024; Kakarla et al., 2022; Lee et al., 2021; Trager et al., 2023; Willson et al., 2021). In inkjet printers, tiny drops of bioink are ejected from the nozzle using thermal or ultrasound pulses and sprayed onto the desired surface. The advantages of this method include high resolution, the ability to print complex structures with high accuracy, and low bioink consumption. However, the disadvantages include the limitation in printing thick structures and the possibility of water evaporation from the bioink (Gu et al., 2020; Mau and Seitz, 2023; Pinto et al., 2023; Wu Z. et al., 2022). Laser printers use a laser to create vapor bubbles in a light-sensitive bioink. These bubbles create pressure and eject bioink droplets from the nozzle. The advantages of this method include high accuracy and the ability to print very fine structures. However, the disadvantages include the high cost and complexity of the system (Wu Z. et al., 2022; Kunwar et al., 2023; Kunwar et al., 2024). Stereolithography printers use a UV laser to polymerize a cell-containing resin. Due to its high speed and high accuracy, this process is suitable for producing complex structures with high details. The advantages of this method include high speed, very high accuracy and the ability to produce complex structures with a lot of detail. However, disadvantages include the limited choice of biomaterials and the need to use photopolymer resins (de Armentia et al., 2022; Desai and Jagtap, 2021; Li et al., 2020; Sengokmen Ozsoz et al., 2024).

Designing and bioprinting a kidney in which its cell lines maintain long-term viability and function is a difficult task. The delicate nature of kidney cells, their specific functional requirements, and the intricate vascular network within the organ make it difficult to maintain their viability and function during the printing process. The high resolution and precision needed to replicate the kidney’s intricate architecture also pose technical hurdles. Additionally, ensuring adequate nutrient and oxygen delivery to cells within the printed structure is crucial but can be challenging due to limitations in diffusion and vascularization. 3D bioprinting has great potential as it can create precise structures, which can lead to better biomimicry (Liu et al., 2021). Two strategies “scaffold-based and scaffold-free” are used for 3D bioprinting (Baptista et al., 2024). 3D bioprinting is still in its early stages and has limitations such as dissociation with the host tissue, angiogenesis, the formation of complex scaffolds, high manufacturing costs compared to other scaffold preparation methods, reproducibility, creation of a functional nephron unit, etc. To solve these limitations, bioprinters are being promoted or new printing methods are being used, such as 4D -Bioprinting (Rahimnejad et al., 2024; Richard et al., 2023; Salah et al., 2020; Shende and Trivedi, 2021; Shokrani et al., 2022).

The direct differentiation of human pluripotent stem cells into kidney organoids brings the prospect of drug screening, disease modeling, and tissue production for kidney replacement, but these applications face problems due to organoid diversity, nephron immaturity, low throughput, and limited scale (Freedman, 2022; Lawlor et al., 2021a; Sander et al., 2020). In a study, extrusion-based 3D bioprinting was used for rapid and high-throughput production of kidney organoids with highly reproducible cell numbers and viability. These organoids were shown to form renal nephrons 20 days after culture. In these nephrons, parts of podocytes, proximal tubules, distal tubules, thick ascending loop of Henle, junctional segments and additional cellular components including endothelial cells and renal stroma were observed (Figure 5B) (Lawlor et al., 2021b). In addition, in this study, they investigated the effect of changing different bioprinting parameters on the characteristics of the resulting organoids in terms of their biological and morphological properties and showed that changing the parameters caused changes such as organoid size, cell number and composition, by modifying the structure. An organoid that significantly increases nephron efficiency per primary cell number. The results showed that the modification of the linear structure of 3D printing has a high nephron number. The authors evaluated cell viability after administration of aminoglycosides, which significantly reduced drug-induced nephrotoxicity. Due to rapid differentiation and loss of key transporters and metabolic enzymes, kidney organoids were more effective than 2D cultures of renal proximal tubule epithelial cells in predicting drug-induced nephrotoxicity (Lin and Will, 2012; Hallman et al., 2008). Finally, they succeeded in creating a kidney patch that contained 4 × 10^5^ cells in a total field of approximately 4.8 mm × 6 mm (Lawlor et al., 2021b). Studies have reported the vascularization and maturation of these organoids after transplantation into the kidney subcapsule of mice (van den Berg et al., 2018). These studies demonstrated that automated extrusion-based bioprinting for kidney organoid production provides improvements in throughput, quality control, scale, and structure, and facilitates *in vitro* and *in vivo* applications of stem cell-derived human kidney tissue.

The final stage of chronic kidney disease (CKD) is end-stage kidney disease (ESKD), in which the kidneys are so damaged that they can no longer perform their vital functions. 3D bioprinting can be used in the treatment of ESKD in regenerative medicine with partial restoration of kidney function. It is worth noting that restoring at least ten percent of kidney function may allow patients with ESKD to escape the harsh conditions of dialysis (Locatelli et al., 2005). To investigate the effect of mesh patch transplantation in the space under the kidney capsule of mice suffering from kidney injury due to unilateral ureteral obstruction, Jo H et al. developed an autologous patch using a bioprinter to study its role in treating ESKD (Jo et al., 2022). In this study, they used two biological essences containing autologous omentum tissue: fibrinogen and thrombin (Figure 5C). After the patch was printed, it was transplanted into rats and renal tubule damage and fibrosis-related gene expression were measured 2 weeks after transplantation. Compared to the group of rats transplanted with the fibrin patch group, a reduction in tubular damage and dysregulation of fibrotic mechanisms was observed in the mesh patch group. One study examined the therapeutic role of renal vascular tubular tissue transplantation in a model of chronic kidney disease (Singh et al., 2020). For this purpose, pig kidneys were first decellularized and lyophilized to produce the matrix from kidney cells. To produce a bioink, the decellularized tissue was then mixed with sodium alginate. Human bone marrow-derived mesenchymal stem cells, human umbilical vein endothelial cells, and renal proximal tubule epithelial cells were used in coaxial 3D bioprinting to create complex single-layer and double-layer hollow structures. After 4 weeks of culture under conditions of vascularized proximal renal tubules, the grafts were transplanted into the subcapsular part of the kidney of immunodeficient mice with a unilateral ureteral obstruction model. Two weeks after transplantation, transplant models with ureteral obstruction showed decreased expression of α-smooth muscle actin and increased expression of aquaporin 1, as well as expression of markers indicative of neovascularization, compared to non-transplanted models.

3D bioprinting offers great potential for kidney regeneration and a solution to the limitations of current therapies for kidney disease. While significant technical and biological challenges remain, ongoing research and technological advances bring us closer to the goal of creating functional, transplantable kidney tissues. Continued interdisciplinary collaboration to realize the full potential of 3D bioprinting in regenerative medicine will be critical to overcoming limitations (Chae and Cho, 2023; Choi et al., 2023; Jamee et al., 2021; Varpe et al., 2024; Zoccali et al., 2024).

## 5 Organ-on-A-chip

**FIGURE 6:**
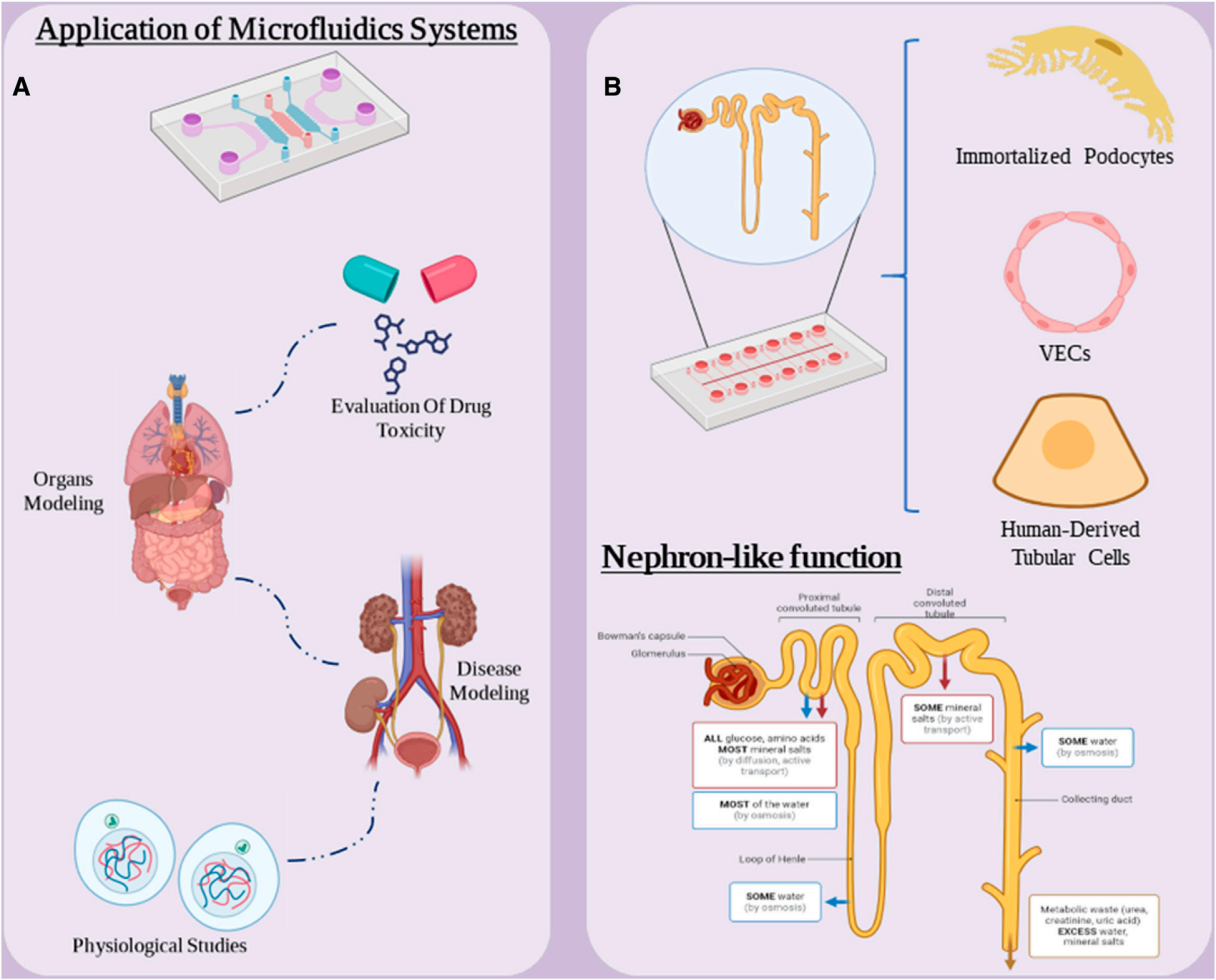
**(A)** Applications of microfluidic systems in medical research. **(B)** “nephron-on-a-chip” components and chip functionality. Figure was created with BioRender.com.

Organ-on-a-chip is an emerging and unique technology in the field of biology and medical technology. This technology allows us to simulate and create miniature models of various organs of the human body for various purposes in the laboratory. These chips contain living cells and an environment that mimics the desired organ tissues (Kumar et al., 2024; Tian et al., 2023). Kidney-on-a-chip technologies have great potential to facilitate the development of kidney models that incorporate key physiological aspects of the tissue microenvironment *in vitro* to study the underlying mechanisms of kidney function and disease, and have also been widely used as a platform for this uses screening for nephrotoxicity (Figure 6A) (Kim and Sung, 2024). Kidney-on-a-chip has the potential to serve as a small, low-cost bridge between very simple *in vitro* cell culture models and expensive and complex animal models, and the manufacturing techniques lend themselves to rapid prototyping and repeatable device design (Halwes et al., 2023). Chips can contain single or multiple cell types in a microchamber or microchannels with or without continuous flow and ECM to create a target tissue-like microenvironment for cell or drug testing. Furthermore, the integration of electrical, electrochemical, and optical sensors into microfluidic devices enables real-time monitoring of cell function, growth rate, and cell monolayer integrity (Tawade and Mastrangeli, 2024).

In 2016, microfluidic-based permeable chips were developed for the first time, in which the proximal renal tubules were completely embedded in an extracellular matrix (Homan et al., 2016). The proximal tubules were surrounded by proximal tubular epithelial cells. These cells ensure that the cell survives and maintains its function for longer than 2 months. A triblock copolymer of polyethylene-polypropylene-polyethylene and thrombin was used to prepare bioink. The prepared model was used to study the mechanisms of drug-induced tubular injury by causing dose-dependent tubular injury using cyclosporine A. The expression results of 33 key proximal tubular epithelial cells after printing and culture showed that these cells are transcriptionally similar to primary kidney proximal tubular epithelial cells. One of the drawbacks of this study is that these model lacks blood vessels and its use in renal reabsorption studies is limited. To address this limitation, a study was conducted in 2019 with the aim of developing a model of vascularized proximal tubules based on microfluidics to study solute reabsorption through vascular tubular exchange (Lin et al., 2019). The results of the markers confirmed the presence of endothelial tissue and, moreover, the perfusion model showed the active reabsorption of albumin and glucose. Furthermore, the ability of this model to simulate the disease by inducing hyperglycemic states and monitoring endothelial cell dysfunction was demonstrated.

The development of chip-based bioengineered kidneys represents a promising advance in the field of renal replacement therapy (RRT) for patients with end-stage renal disease (ESRD). Microfluidics technology has played an important role in the development of chip-based implantable artificial kidneys (IAKs). Designed to be more efficient than traditional dialysis in terms of waste removal and nutrient retention. Microfluidic artificial kidneys have demonstrated higher permeation efficiency *in vitro*, and *in vivo* experiments have demonstrated their potential to improve kidney function in models of kidney failure, which is important for maintaining the function and longevity of the bioengineered kidney. The microchip filter contains a framework that mimics the structure of a kidney cell membrane, thereby regulating the separation of metabolic waste and nutrients in the blood. Chip-based IACs offer several advantages over traditional fiber dialyzers. Because they are compact, they no longer require dialysis and are more economical. They also use the patient’s blood pressure to direct blood through the filter to reduce the risk of blood clots. In chip-based IACs, human-derived biomaterials are used to minimize the risk of immune rejection, and ECM components in these chips reduce the absorption of added proteins (Van Dang et al., 2019). The effectiveness of human trials evaluating chip-based IACs may completely replace this technology with traditional and costly dialysis methods.

Nephron on a chip consists of a glomerular component consisting of GECs and podocytes and a tubular section consisting of VECs and PTECs, which are separated from each other. The two are separated by a porous membrane into separate channels for blood and urine. Using parallel microchannels to mimic renal blood flow and filtration drainage, these chips simulate the glomerular barrier and load-selective functions within the renal corpuscle and in the “proximal tubule” the model reabsorbs glucose and releases para-amino-hippuric acid. This chip is a suitable nephron model for studying the pathophysiological mechanisms of AKI and drug nephrotoxicity. In one study, glomerulus and proximal tubule microchips were designed and integrated into a multilayer culture system to construct a nephron model *in vitro* (Figure 6B) (Zhang and Mahler, 2023). This T-junction model simulates serum protein filtration, glucose reabsorption and creatinine secretion. This chip consists of immortalized podocytes, VECs and human-derived tubular cells. They used this model to study cell connectivity disorders, drug-induced renal toxicity, filtration and reabsorption. They used cisplatin and adriamycin to evaluate the performance of this model. The results showed that this model replicated some in vivo-like functions under basal and drug-induced conditions and provided a new prototype for clinical studies, highlighting the potential of Nephron-on-a-Chip as a promising simulation model.

Significant advancements in tissue engineering have led to the development of kidney-on-a-chip systems. However, one of the primary challenges in this field has been the accurate reconstruction of the kidney’s complex architecture, particularly the endothelial-epithelial barrier. This barrier plays a crucial role in blood filtration and nutrient reabsorption. In a recent study, researchers have successfully developed a highly customizable kidney-on-a-chip system capable of recreating this barrier with exceptional precision. The system utilizes a two-layered human renal vascular-tubular unit, activated by a thin collagen membrane. Endothelial and epithelial cells spontaneously remodel this membrane into a barrier with a near-native thickness, composed of native basement membrane proteins. This barrier provides sufficient mechanical integrity for media flow and perfusion and mimics essential kidney functions such as albumin and glucose reabsorption (Rayner et al., 2018).

To create and enhance biomimetic chips, a combination of methods such as adding biosensors or structures like electrospun sheets is employed in their design (Ngocho et al., 2024). In one study, a kidney glomerulus was engineered on a chip to simulate glomerular morphogenesis and barrier function using an electrospun ultrathin biomimetic membrane and human induced pluripotent stem cells (Mou et al., 2024). At first, a 5% aqueous solution of silk fibroin (SF) was dialyzed against a 90% aqueous solution of polyethylene glycol, and then reconstituted in ultrapure water to achieve an 8% SF solution. The resulting solution was mixed with a 10% aqueous solution of PEO and electrospun at a voltage of 11 kV. PDMS was cast in pre-prepared molds to create ready-made PDMS chips. The designed chip had two separate parts that needed to be joined. Electrospun membranes measuring 5 cm by 0.5 cm were pre-sealed onto the lower chip, which was coated with a PDMS pre-polymer mixture adhesive containing Sylgard 184 elastomer and a curing agent. The upper PDMS chip coated with PDMS adhesive was then attached to the lower SF/PDMS chip. After joining the chips, laminin-511 was added to the upper and lower channels of the chip and then incubated at 37°C. Evaluation results of the chip revealed a near-epithelial-endothelial tissue interface that mimicked the selective molecular filtration function of healthy and diseased kidneys, indicating a dynamic tissue engineering platform for modeling human kidney morphogenesis and specific function. Additionally, since glomerular basement membrane thickening is a hallmark of several glomerular disorders such as membranous nephropathy, understanding how membrane thickness affects podocyte foot processes can improve understanding of kidney disease mechanisms and aid in identifying therapeutic targets in these disorders (Mou et al., 2024).

Research into the health of astronauts is of paramount importance. These studies are not only crucial for ensuring the wellbeing of astronauts on long-duration missions but also provide invaluable insights into human physiology and disease. By examining the physiological changes that occur in microgravity, scientists can develop novel treatments for conditions like kidney damage and improve life support systems for future space missions. In essence, research on astronaut health benefits both space explorers and humanity as a whole (Minoretti et al., 2024; Pandith et al., 2023). The International Space Station (ISS) has offered unique insights into the effects of microgravity on human physiology. Studies on astronauts have revealed a range of pathological changes, including alterations in kidney function. To better understand the underlying mechanisms and develop strategies for long-duration spaceflight, researchers have investigated the impact of microgravity on kidney cells cultured in microphysiological systems (Kelly et al., 2023; Lidberg et al., 2024). A key area of focus has been the response of proximal tubule epithelial cells (PTECs) to serum exposure and vitamin D metabolism. These cells play a crucial role in blood filtration and electrolyte balance. Previous studies have shown that serum exposure can induce toxicity and inflammation in PTECs, while vitamin D is essential for maintaining kidney health. Recent research conducted aboard the ISS has examined how microgravity affects these processes in PTECs. By culturing PTECs in a microphysiological system and exposing them to human serum or vitamin D under microgravity conditions, researchers have been able to identify potential mechanisms of change. For instance, studies have investigated the expression of genes involved in toxicity and inflammation, as well as the formation of vitamin D metabolites. Overall, these studies have provided valuable information about the impact of microgravity on kidney function. While short-term exposure to microgravity does not appear to significantly alter PTEC vitamin D metabolism or their response to serum, the long-term effects of microgravity remain unknown. As future spaceflight missions become longer, it will be crucial to continue investigating the effects of this unique environment on the kidney and develop strategies to mitigate any adverse consequences (Lidberg et al., 2024).

As previously mentioned, kidney on a chip is an emerging technology in the field of regenerative medicine that allows the study of kidney function, kidney disease and the effects of medications in a controlled environment. For all the advantages of this technology, like any other technology, there are limitations (Huang W. et al., 2024). The challenges leading to kidney-on-a-chip limitations fall into several categories, including the complexity of kidney function, cell limitations, modeling limitations, high cost, and technical complexity (Wang J. et al., 2022). Kidneys are responsible for interorgan interactions and very complex tasks (including filtering blood, regulating blood pressure, producing hormones, and maintaining electrolyte balance) (Fularski et al., 2024). Simulating and designing all of these functions on one chip is a major challenge. Furthermore, kidney function changes over time and in response to different stimuli, and simulating these dynamic changes requires more complex models. In addition, due to the mechanobiological properties of the surface, the cells in the chip can change their phenotype and their behavior can differ from that of the cells in the body (Pivonka et al., 2024; Wang D. et al., 2022). Despite these limitations, kidney-on-a-chip technology has very great transformative potential in the field of medicine, and with the advancement of technology and research, the integration and application of various sciences, and the improvement of simulation knowledge, there are many more of these Limitations will be overcome in the future (Huang T. et al., 2024). Table 1 provides an overview of the studies carried out in the field of kidney tissue engineering with regard to the mentioned technology.

**TABLE 1:** An overview of the studies conducted in the field of Kidney tissue engineering.

******	Material	Target	Results	Ref
Hydrogel	Oxidized Alginate (Oxi-Alg)	Controlled and biomimetic environment for organoid encapsulation	Rigid hydrogel encapsulation resulted in the absence of certain renal cell types and markers of epithelial-mesenchymal transition (EMT), whereas encapsulation in soft hydrogels resulted in all major renal compartments, less fibrosis, or EMT-related proteins	Ruiter et al., 2022
	Alginate and Thiol-ene	Modulation of kidney organoids by ECM	Decreased expression of collagen type 1a1 No change in organoid structural morphology Increased expression of specific collagen subtypes associated with renal fibrosis	Geuens et al. (2021)
	Decellularization ECM (dECM) from porcine and human renal cortex	Supporting the production of kidney organoids and promoting angiogenesis	The resulting renal organoids show the characteristics of renal differentiation and the formation of an endogenous Renal differentiation of hPSCs	Garreta Bahima et al. (2024)
	GelMA	Production of human iPSC (hiPSC)-derived kidney organoids	Direct differentiation of human induced pluripotent stem cells (hiPSCs) into kidney organoids and maturation in mechanical self-assembling peptide hydrogels (SAPHs)	Krupa et al. (2024)
	UPy-Cy5 andUPy-GRGDS-Cy5	Responsive mechanical nano-environment with composite cell adhesive properties	Regulation of glomerulogenesis within kidney organoids	Sprang et al. (2024)
	self-assembling peptide hydrogels (SAPHs)	Growth and differentiation of human induced pluripotent stem cell (hiPSC)-derived kidney organoids	The resulting organoids contain complex structures Nephron cell types were observed in Transwell-derived organoids	Treacy et al. (2022)
	Alginate	Regulation of nephrogenesis in kidney organoids	Formation of tubular nephron complex segments in kidney organoids	Nerger et al. (2024)
Electrospinning	Silk fibroin	Biomimetic membrane to support the differentiation and maturation of kidney epithelium	Differentiation of hiPS cells into post-mitotic kidney glomerular podocytes on membrane	Mou et al. (2022b)
	laminin and polycaprolactone	Synthesis of hybrid scaffolds for the growth and proliferation of kidney cells	Maintaining the survival of human kidney epithelial cells up to 3 weeks after culture on the scaffold	Başkapan and Callanan (2021)
	Poly (lactic acid)	A suitable substrate for the culture of rat primary kidney cells	the scaffolds were capable of sustaining a multi-population of kidney cells, determined by the presence of: aquaporin-1 (proximal tubules), aquaporin-2 (collecting ducts), synaptopodin (glomerular epithelia) and von Willebrand factor (glomerular endothelia cells), viability of cells appeared to be unaffected by fibre diameter	Burton and Callanan (2018)
3D Printing	dECM, sodium alginate, pluronic	Proximal tubule model	Cells bioprinted in different concentric configurations; Function demonstrated	Singh et al. (2020)
	decellularized kidney ECM (dKECM), agarose microparticle	explore the potential of using unmodified decellularized kidney extracellular matrix (dKECM) as a bioink	Effective bioprinting, High cell viability, Complex structure formation, ability to provide tissue-specific cues	Sobreiro-Almeida et al. (2021)
	dECM, Methacrylation	create clinically applicable kidney tissue constructs	structural and functional characteristics similar to native kidney tissue, Cell viability and maturation	Ali et al. (2019)
	gelatin-fibrin ECM	Bioprinting of complex renal proximal tubules	The complex tubular architecture is surrounded by epithelial cells of the proximal tubule and is actively perfused through the open lumen	Homan et al. (2016)
	Biomaterial ink free	Kidney organoid model for drug testing	Patterning with a clear influence on the differentiation and maturation of organoids	Lawlor et al. (2021b)
Organ-On-A-Chip	Alginate, pectin and Gelatin + PDMS	Kidney tubulointerstitium	Multiple configurations in core shell format	Addario et al. (2020)
	silk fibroin + PDMS	Simulation of glomerular morphogenesis and barrier function	Epithelial-endothelial cell interface and selective molecular filtration function	Mou et al. (2024)
	PDMS	Personalized glomerular chip system	Reconstruction of intercellular junctions and hypertrophy	Roye et al. (2021)
	gelatin-fibrin ECM + PDMS	Vascular and permeable renal organoid	Migration of renal organoids through the ECM towards the great vessel (where they form lumen-on-lumen anastomoses)	Kroll et al. (2024)
	gelatin-fibrin ECM + PDMS	Organoid-on-a-chip kidney model with immune infiltration	Peripheral blood mononuclear cells (PBMCs) were co-cultured with non-targeting (control) or targeting TCB-based tool compounds	Kroll et al. (2023)
	CHIPit™ and comPLATE™	Creating a kidney organoid-vasculature interaction model	Migration and proliferation of human umbilical vein endothelial cells (HUVECs) The formation of an open lumen similar to vessels	Menéndez et al. (2022)
	Fibrinogen and Gelatin + Polycarbonate	Creation of a permeable 3D proximal tubule model	Significant positive regulation of organic cation (OCT2) and organic anion (OAT1/3) transporters (leading to improved drug absorption)	Aceves et al. (2022)
	Collagen Membrane + PDMS	Reconstructing the Human Renal Vascular-Tubular Unit	provides sufficient mechanical integrity for media flow and perfusion and mimics essential kidney functions such as albumin and glucose reabsorption	Rayner et al. (2018)

## 6 Future perspectives

Given the significant advances in the field of kidney tissue engineering and the high potential of this field in the treatment of kidney diseases, various research paths are foreseeable in the future. Among these possibilities, it is possible to design and fabricate scaffolds that accurately mimic the extracellular structure of the kidney, including the creation of chemical and mechanical gradients that can lead to improved cell differentiation and tissue performance. A more detailed study of the molecular mechanisms that control the differentiation of stem cells into kidney cells and the genetic engineering of these cells to increase their efficiency and stability are also important goals of future research. Creating models of human-like kidney disease allows for more accurate and comprehensive evaluation of tissue engineering-based therapies. To this end, organ-on-chip systems can help reduce animal testing, as designing microfluidic systems with the ability to accurately simulate the physiological environment of the kidney and precisely control environmental parameters can help better control pathogenic processes understand and develop new treatment methods. The combination of different tissue engineering methods such as 3D bioprinting, electrospinning and hydrogels, as well as the use of engineered cells and microfluidic systems can lead to the creation of more complex and efficient engineered tissues.

In addition to the cases mentioned, future research in the field of kidney tissue engineering should focus on things such as developing more accurate assessment methods for engineered tissue, studying the long-term effects of transplantation of engineered tissue, and developing combined treatment approaches. Due to the complexity of kidney structure and function, it remains a major challenge to create a completely artificial kidney that can perform all the functions of a normal kidney. However, recent advances in the field of tissue engineering are promising and show that this field can play an important role in the treatment of kidney diseases.

## 7 Conclusion

Renal tissue engineering has been proposed as a new approach to treating kidney diseases with the aim of regenerating and repairing damaged kidney tissue. This review discussed recent advances in various constructs used in kidney tissue engineering, including hydrogels, electrospun scaffolds, 3D bioprinting, and microfluidic systems. Hydrogels are considered one of the most popular scaffolds in kidney tissue engineering due to their high biocompatibility and the ability to customize their physical and chemical properties. These structures create an environment similar to the extracellular matrix and support the growth and proliferation of kidney cells. By creating nanofibrous structures, electrospun scaffolds mimic an environment similar to the kidney’s natural tissue, providing the ability to control cells and regulate their behavior. These scaffolds can also be loaded with growth factors and drugs to create a suitable environment for tissue repair. As an emerging technology, 3D bioprinting enables the production of complex scaffolds with arbitrary geometries and controlled cell distribution. This technology has high potential for producing artificial tissue with the same structure and function as natural kidney tissue. Microfluidic systems offer the possibility of creating microfluidic environments with precise control of physical and chemical conditions. These systems can be used to study biological processes at the cellular and tissue level and evaluate the effectiveness of new treatments. Despite significant advances in the field of kidney tissue engineering, challenges still exist, including the complexity of the kidney structure, the need for sufficient cell sources, and issues of safety and clinical efficacy. Future research should focus on the development of smarter scaffolds, the use of stem cells and genetic engineering, and the clinical evaluation of engineered constructs. In general, kidney tissue engineering using structures such as hydrogels, electrospun scaffolds, 3D bioprinting, and microfluidic systems has high potential for treating kidney diseases and improving patients’ quality of life.
